# Targeting lemurs against cancer metastasis

**DOI:** 10.18632/oncotarget.2271

**Published:** 2014-07-25

**Authors:** Yichen Xu, Hua Zhang, Georgios Giamas

**Affiliations:** Department of Surgery and Cancer, Division of Cancer, Imperial College London, Hammersmith Hospital Campus, Du Cane Road, London W12 ONN, UK

Breast cancer is the most frequently diagnosed cancer and the leading cause of mortality in females worldwide. Despite the great improvement in survival rates the last two decades, relapsed disease with metastasis still remains the most poorly understood aspect of cancer pathogenesis [1]. Consequently, there is a growing need to identify so-called new ‘players’ implicated in this process. Lemur tyrosine kinase 3 (LMTK3), a member of the receptor tyrosine kinase (RTK) family, has been previously identified as a central protein involved in breast cancer growth and endocrine resistance and it has also been subjected to Darwinian positive selection as described [2-5]. Moreover, increased LMTK3 expression associates with poor overall and disease-free survival, indicative of a potential involvement of LMTK3 in breast cancer progression.

Cell migration and invasion are the early steps of the metastatic process controlled by cellular signals that stimulate changes in cell adhesion and cytoskeleton formation [6]. We have now reported that LMTK3 is highly expressed in triple negative breast cancers and it is implicated in actin cytoskeleton remodeling, focal adhesion formation and increased cell motility [7]. *In vitro* experiments showed that overexpression of LMTK3 accelerates the dispersion and invasion of breast cancer cells towards the matrigel and collagen, while it increases actin protrusions and focal adhesion formations on the edge of migrating cells. To address the underlying molecular mechanisms, a stable isotope labeling by amino acids in cell culture (SILAC)-based proteomics analysis was implemented to investigate the downstream components of LMTK3-regulated cascade. Interestingly, integrin subunits α5 (ITGA5) and β1 (ITGB1), central receptors in metastatic signal relay, were positively regulated by LMTK3, while high LMTK3 levels were also correlated with increased ITGB1 expression in breast cancer patient samples. In support of the role of LMTK3 in metastatic progression, a proof-of-concept *in vivo* metastatic mouse model was generated, after injecting parental T47D breast cancer cells and T47D cells overexpressing LMTK3 into the mammary fat pads of female NOD/SCID mice. Our preliminary results revealed that only the T47D-LMTK3 mice developed metastasis in the mammary fat pad after 7 weeks, suggesting the contribution of LMTK3 in metastatic potential (unpublished data).

Similar to other RTKs, LMTK3 can directly interact with the adaptor protein GRB2, activate RAS-GTPase family members subsequently leading to upregulated serum response factor (SRF) activity, resulting in increased *ITGA5* and *ITGB1* gene transcription. A set of gain- and loss-of-function experiments showed that LMTK3 could stimulate SRF indirectly through its action on cell division control protein 42 homolog (CDC42). However, other alternative mechanisms merit investigation, including the possibility that LMTK3 might activate SRF through direct phosphorylation. In support of this hypothesis, *in silico* analysis revealed potential phosphorylation sites of SRF targeted by LMTK3 that are currently under examination. Moreover, the topology of LMTK3 (cytoplasmic, nuclear and to a lesser degree in the cell membrane), implies that LMTK3 may be capable of shuttling between subcellular compartments upon specific stimuli (i.e. posttranslational modifications) and other cellular conditions. Therefore, understanding the exact role(s) of LMTK3 in different cellular compartments is imperative. In order to characterize substrates phosphorylated by LMTK3, a high-throughput *in vitro* kinase assay screen is in progress that will further help us uncover the signaling pathways that LMTK3 is implicated in and decipher its contribution in disease.

**Fig 1:**
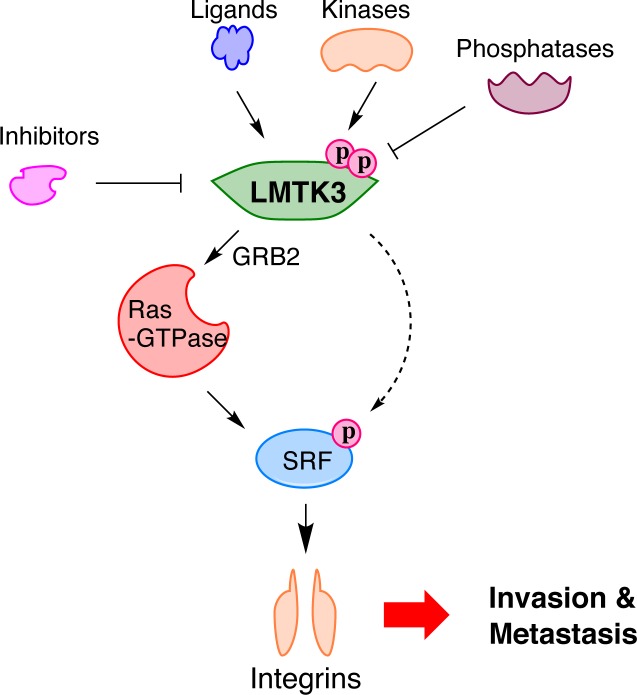
Proposed model of LMTK3 action in cancer metastasis In response to various stimuli (i.e ligands binding, regulation by kinases or phosphatases, etc) LMTK3 activates Ras-GTPase family through GRB2, enhancing the activity of the SRF transcription factor. In parallel, LMTK3 might activate SRF through direct phosphorylation that eventually results in the activation of integrins transcription, which subsequently leads to increased cancer cell invasion and metastasis.

Furthermore, elucidating the upstream mechanisms of LMTK3-regulation is also essential. Till now, there have been no studies regarding the existence of any LMTK3-specific ligand(s) or other proteins (i.e. kinases, phosphatases, etc.) that can directly activate/inactivate the catalytic activity of LMTK3. Unraveling the upstream signaling and cross-talk pathways of LMTK3 would greatly improve our understanding of the underlying mechanisms that contribute to the regulation of LMTK3 and its involvement in invasion and metastasis (Fig 1).

In aggregate, based on our results so far, LMTK3 appears to be a promising new target against breast cancer progression and metastasis. Additional work is required to broaden our knowledge of LMTK3 functions at molecular and cellular levels. In parallel, a drug screening program will facilitate the identification of specific LMTK3 inhibitors, which could eventually aid to tackle breast cancer metastasis and endocrine resistance.
